# Outcomes and Complications of Mobile Inferior Pedicle Breast Reduction as a Modification of Inferior Pedicle

**DOI:** 10.1007/s00266-025-05601-5

**Published:** 2026-01-06

**Authors:** Nazım Gümüş

**Affiliations:** Department of Plastic, Reconstructive and Aesthetic Surgery, Mersin University Medical Faculty, Mersin, Turkey

**Keywords:** Mobile inferior pedicle, Chest wall flap, Advancement, Bottoming out, Pseudoptosis

## Abstract

**Background:**

This study evaluates a modified breast reduction technique, the mobile inferior pedicle (MIP), designed to overcome the drawbacks of the traditional inferior pedicle (IP) method, such as bottoming out, poor projection, and areolar malposition.

**Materials and Methods:**

A total of 70 patients were included in this retrospective review study. The key modification involved incising the inferior pedicle down to the pectoral fascia, mobilizing it superiorly, and securing it in the upper pole to enhance projection and shape.

**Results:**

Outcomes demonstrated successful volume reduction and improved breast projection. The nipple was positioned at the breast apex in 87% of cases. The overall complication rate was 22.8%, with most issues managed outpatient. Only two cases of partial nipple necrosis were reported. Follow-up time ranged from 9 to 39 months with a mean of 23.37 months. Critically, symptoms like shoulder, neck, and back pain were significantly reduced, and patient satisfaction was high. The rate of bottoming out was observed in 12.8% of breasts.

**Conclusion:**

In conclusion, the MIP technique effectively addresses common criticisms of the IP method. By improving breast shape and projection while maintaining a manageable complication profile, it presents a strong and valuable alternative for achieving aesthetically pleasing and durable results in breast reduction surgery.

**Level of Evidence IV:**

This journal requires that authors assign a level of evidence to each article. For a full description of these Evidence-Based Medicine ratings, please refer to the Table of Contents or the online Instructions to Authors www.springer.com/00266.

## Introduction

Reduction mammaplasty is a common and essential procedure for removing excess breast tissue, reducing volume, and reshaping the breast. Maintaining a robust blood supply to the nipple–areolar complex (NAC) is imperative for ensuring the safety and success of the operation. Numerous surgical techniques, each based on a different vascular pedicle, have been described and popularized. Among these, the inferior pedicle technique with a wise-pattern incision (IP) has been considered the most reliable and widely used method since its description by Ribeiro in 1975 [1]. This technique is versatile, applicable to a wide range of breast sizes, and is particularly well suited for larger resections. It boasts a low complication rate and a high degree of preservation for both nipple sensation and lactation potential. Further advantages include its applicability to patients with a long sternal notch-to-nipple distance and its short learning curve [1, 2].

Despite its popularity, the IP technique is frequently criticized for several shortcomings. These include the development of pseudoptosis (“bottoming out”) due to the pedicle’s tendency to descend inferiorly, areolar malposition, hypertrophic scarring, boxy breast contours, a lengthy operative time, wound dehiscence at the T-junction, and poor long-term projection [2–6]. These limitations have led to the development of numerous modifications, most aiming to prevent the inferior descent of the pedicle [7–15]. A recent modification involves dividing the inferior pedicle completely from the inframammary fold and mobilizing it superiorly. By incising the pedicle’s base down to the pectoral fascia, the pedicle is converted into a mobile, chest wall-based flap, gaining significant mobility [16].

In this study, we term this modification the ’mobile inferior pedicle’ (MIP) technique to distinguish it from the conventional inferior pedicle (IP) procedure. We evaluate the long-term outcomes of patients who underwent breast reduction using the MIP technique and discuss its efficacy in overcoming the well-documented complications associated with the standard IP approach, based on the literature findings of inferior pedicle.

## Patients and Methods

### Operative Technique

**Fig. 1:**
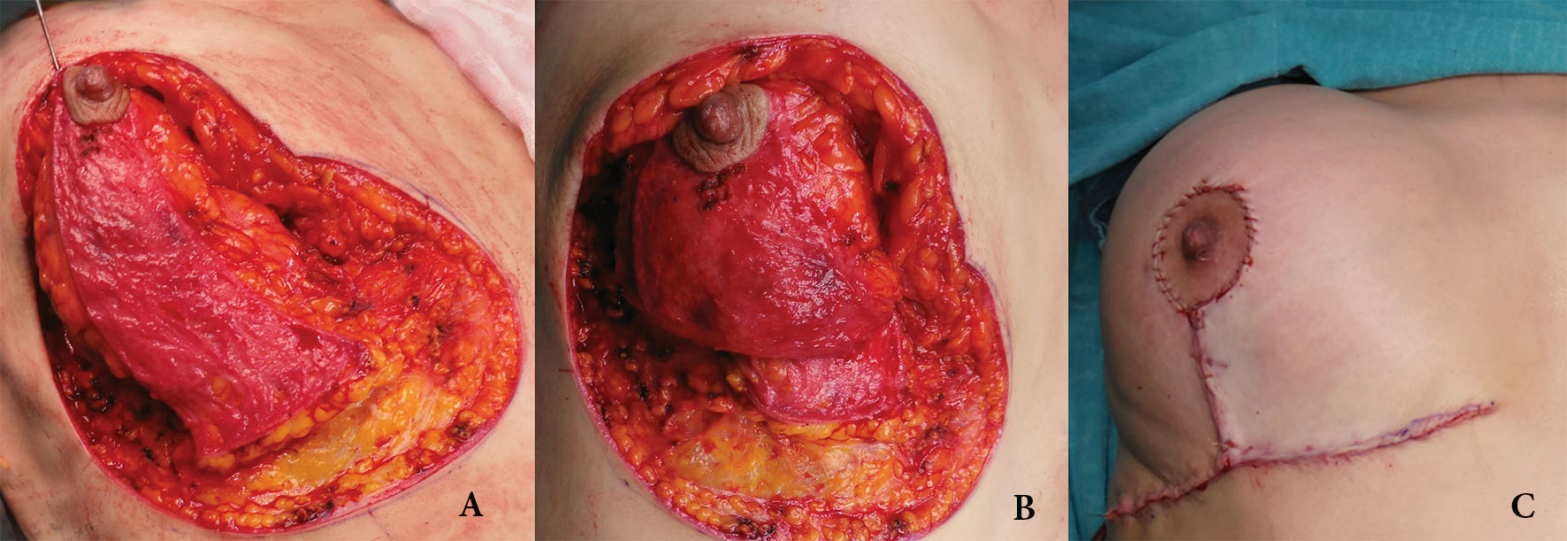
**A** Appearance after dissection of the pedicle that was incised straight down to the pectoralis fascia at the base area. Note that there was not any dermal or glandular attachment to the inframammary fold. **B** Dermal stitches that were placed between dermis and pectoral muscle after mobilization of the pedicle. **C** Intraoperative breast shape just after skin closure

The reduction mammaplasty procedure utilizing the mobile inferior pedicle (MIP) shares similar preoperative markings and surgical steps with the conventional inferior pedicle technique, making it a highly reproducible procedure with a short learning curve. The MIP surgical technique used in this study closely followed the procedure described by Gümüş [17]. Preoperative markings were made with the patient in a standing position. These included the breast meridians, the inframammary folds (IMF), and the new nipple position. The provisional nipple site was marked at the level of the IMF, approximately 21–22 cm from the sternal notch. Skin markings were then completed using a standard inverted-T (Wise) pattern, with the vertical limbs (the nipple-to-IMF distance) standardized to 8 cm for all patients. The final nipple position was determined intraoperatively to optimize symmetry and projection, and therefore, a periareolar marking was not made preoperatively. An inferior pedicle was designed with a width of 8–10 cm, preserving a 1 cm cuff of tissue around the areola (Fig. 1).

The procedure began with an incision around the areola to create a 42 mm-diameter opening. The pedicle was then completely de-epithelialized. The dermis and breast tissue of the pedicle were incised straight down to the prepectoral fascia; the fascia itself was neither opened nor detached. Crucially, no dissection was performed underneath the pedicle (Fig. 1A). During the excision of the lateral and medial pillars, the prepectoral fascia was left intact. Skin flaps were maintained at a thickness of at least 2 cm to preserve their vascular supply. To enhance pedicle perfusion, the deep connections of the pedicle to Würinger’s horizontal septum were carefully preserved during glandular excision. A subglandular pocket was created in the upper pole of the breast. This dissection was performed from the mid-clavicular line under the superior skin flap, extending up to the level of the second rib. Subsequently, the base of the inferior pedicle was incised down to the pectoralis fascia, severing all dermal and glandular attachments to the inframammary fold. This critical step converted the inferior pedicle into a mobile, chest wall-based flap. This new mobility allowed the flap to be transposed superiorly to fill the central and upper portions of the breast, enhancing projection. The flap was then secured in this new position. Three fixation sutures were placed between the deep surface of the flap (below the areola) and the pectoralis muscle at the level of the second intercostal space using a long-lasting PDS suture. Two additional symmetrical sutures were placed to further anchor the flap to the pectoral muscle, to protect long-term breast shape and avoid possible sagging (Fig. 1B). The skin flaps were redraped over the newly shaped breast mound and closed in a standard fashion. The final position for the nipple–areola complex (NAC) was determined on the vertical suture line, 5.5 cm from the T-junction. The NAC was then inset and sutured into place (Fig. 1C).

### Study Design

**Fig. 2:**
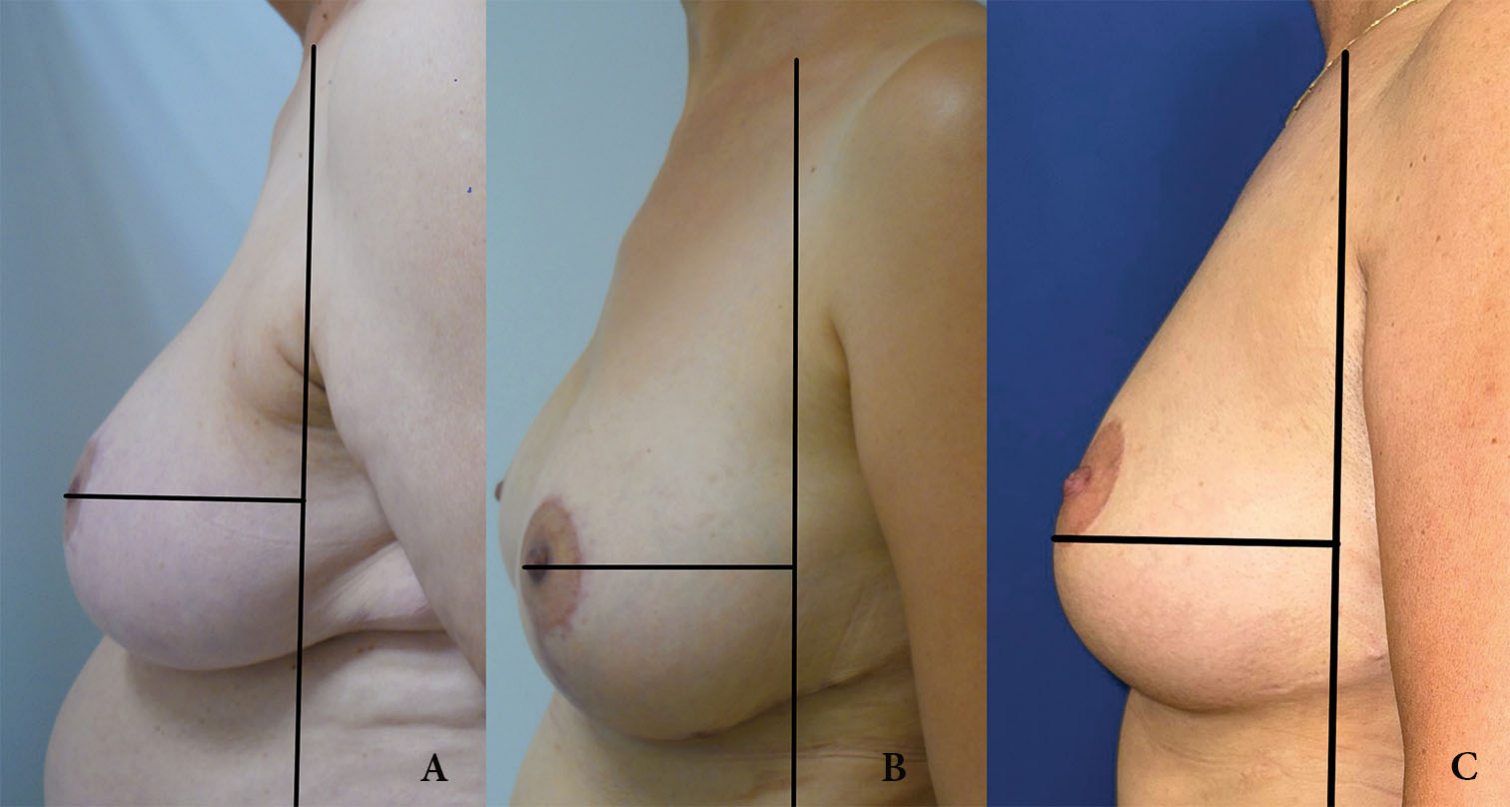
**A** Normal breast view on which the ratio of upper breast area to lower breast area is nearly equal on lateral images. **B** View of perky breast consists of more upper breast area than lower breast area. **C** Bottoming out appearance shows the nipple level that is located upper the apex of the breast

A total of 70 consecutive patients who underwent bilateral breast reduction using the MIP technique between July 2017 and June 2023 were included in this retrospective study. Following approval by the university’s ethics committee, a chart review was conducted to extract patient demographics, medical comorbidities, smoking status, operative details, follow-up duration, and postoperative adverse events. Complications were categorized as minor or major. Minor complications included minor hematoma, seroma, skin flap necrosis, wound dehiscence, fat necrosis, and infections. Major complications were defined as major infections, severe hematoma, and nipple loss. Documented preoperative comorbidities included obesity, diabetes mellitus, coronary artery disease, hypertension, and smoking. Written informed consent was obtained from all participants. The breasts were divided into two groups (cutoff at 700g), to understand whether there was a significant difference in complications between the two groups. Also, the breasts were divided into another two groups: smokers and non-smokers to understand whether there was a significant difference in complications. Subgroup analysis was made statistically. Standardized preoperative and final postoperative follow-up photographs were taken for analysis using the photographic evaluation method described by Swanson [9, 18]. The lateral view photographs were used to assess the long-term effectiveness of the technique on breast appearance, specifically breast projection, upper pole fullness, and the breast parenchymal ratio (calculated as the ratio of the upper breast area to the lower breast area on lateral images). On the lateral photographs, a vertical line was drawn at the posterior breast margin. A horizontal line was then extended from this vertical line to the most anterior projection of the breast, indicating the point of maximum projection and the ideal nipple level. The areas above and below this horizontal line were compared to determine the upper and lower pole proportions, thereby quantifying upper pole projection and identifying bottoming out. Following a visual inspection of the photographs, the number and rate of breasts with a normal/perky upper pole, ptosis, or a bottomed-out appearance in the final postoperative lateral views were determined (Fig. 2). Evaluation of lateral postoperative photographs was made by three independent plastic surgeons. Photographs of each patient were presented to them on a computer screen. The presentation was performed using Power Point, Microsoft Office software. The surgeons were asked to evaluate the result as “normal upper pole”, “perky upper pole”, “ptosis”, or “bottomed-out appearance”.

Patients were asked to complete the BREAST-Q Reduction/Mastopexy module preoperatively and postoperatively. Responses were recorded on a Likert scale to evaluate changes in satisfaction levels. Associations between preoperative variables and outcomes were compared statistically using Chi-square tests, Wilcoxon tests, and Fisher’s exact test. A *p* value of less than 0.05 was considered statistically significant.

## Results

Patient ages ranged from 20 to 71 years, with a mean age of 45.33 years. They presented with mild-to-severe breast hypertrophy. The average weight of tissue excised was 727.88 g (range: 80–1800 g) for the right breast and 699.62 g (range: 80–1800 g) for the left breast. The mean follow-up time was 23, 37 months, ranging from 9 to 39 months. Twenty-seven patients had preoperative comorbidities, including diabetes mellitus, hypertension, and smoking; regular tobacco use was the most frequent comorbidity (Table 1). In the early postoperative period, the procedure successfully achieved breast volume reduction, increased projection, elevated the lower pole, and positioned the nipple at the breast apex. The breast parenchymal ratio also increased by an average of 0.5. Surgical complications were observed in 25 patients (17.8%) and included delayed healing, wound dehiscence, fat necrosis, infection, cyst formation, hypertrophic scarring, and nipple loss (Table 2). Two cases of fat necrosis were observed. One was a 2 × 2 cm lesion located superior to the areola at the midline, and the other was a 2 × 3 cm lesion in the lower lateral quadrant of the breast. Two patients with wound infections required hospitalization for 5 days of intravenous antibiotic therapy. The reason for these infections may be diabetes in one patient and smoke in another. All other complications were managed conservatively on an outpatient basis. There was one case of partial and one case of total nipple loss; both occurred in patients who were heavy, regular tobacco users. Reduced nipple sensation was reported by three patients initially, but sensation returned to normal within 6 months postoperatively.

**Table 1:** Demographic data of the patients

Characteristic	Value (%)
Mean age ± SD, years	45,33 ± 12,1
Mean body mass index ± SD, kg/m^2^	28,3 ± 5,4
Mean resection weight right breast ± SD, g	727,88 ± 388,75
Mean resection weight left breast ± SD, g	699,62 ± 403,6
No. of smokers	14(20)
Mean follow-up ± SD, months	23,37 ± 8,87
Diabetes	6(8,5)
Hypertension	7(10)
No. of breasts	140

**Table 2:** Complications of breast reductions with MIP technique

Complications	Overall (140 breasts) No. %		Excision ≥ 700 gr (66 breasts)* No. %		Excision < 700 gr (74 breasts) No. %	
Nipple necrosis	2	1,4	1	1,5	1	1,3
Delayed healing	5	3,5	3	4,5	2	2,6
Wound dehiscence	6	4,4	3	4,5	3	3,9
Fat necrosis	2	1,4	1	1,5	1	1,3
Wound infection	4	2,8	3	4,5	1	1,3
Hypertrophic scarring	2	1,4	1	1,5	1	1,3
Revision	4	2,8	3	4,5	1	1,3
Temporary loss of nipple sensation	3	2,2	1	1,5	2	2,6
Cyst formation	4	2,8	3	4,5	1	1,3
Hematoma	0	0	0	0	0	0
Seroma	0	0	0	0	0	0
Total	32	22,8	19	28,5	13	16,9

*: The large excision group (≥ 700g) showed a higher overall complication rate compared to the smaller excision group. However, this difference was not statistically significant (*χ*^2^ = 2.84, *p* = 0.092).

In the subgroup analyses, the large excision group (≥700g) showed a higher overall complication rate (28.5%) compared to the smaller excision group (16.9%). However, this difference was not statistically significant (*χ*^2^ = 2.84, *p* = 0.092). While the large excision group showed a numerically higher complication rate across most categories, the difference did not reach statistical significance. This suggests that excision weight alone may not be the primary determinant of complication risk in this patient population.

No significant difference was found in overall complication rates between smokers and non-smokers (*p* > 0.05). However, smokers demonstrated higher incidence of nipple necrosis (*p* < 0.05). Smokers appear to be at higher risk for nipple necrosis. The small sample size of smokers (*n* = 28) limits the statistical power to detect differences, particularly for rare complications (Table 3).

**Table 3:** Complications of breast reduction due to tobacco use

Complications	Overall (140 breasts) No. %		Smokers (28 breasts)* No. %		Non-smokers (142 breasts)* No. %	
Nipple necrosis**	2	1,4	2	7,1	0	0
Delayed healing	5	3,5	2	7,1	3	2,1
Wound dehiscence	6	4,4	1	3,5	5	3,5
Fat necrosis	2	1,4	0	0	2	1,4
Wound infection	4	2,8	1	3,5	3	2,1
Hypertrophic scarring	2	1,4	0	0	2	1,4
Revision	4	2,8	0	0	4	2,8
Temporary loss of nipple sensation	3	2,2	0	0	3	2,1
Cyst formation	4	2,8	0	0	4	2,8
Hematoma	0	0	0	0	0	0
Seroma	0	0	0	0	0	0
Total	32	22,8	6	21,2	26	18,2

*: There was no statistically significant difference in overall complication rates between smokers and non-smokers (*χ*^2^ = 0.16, *p* = 0.692). **: There was a statistically significant association between smoking and nipple necrosis (Fisher’s Exact Test *p* = 0.043).

**Fig. 3:**
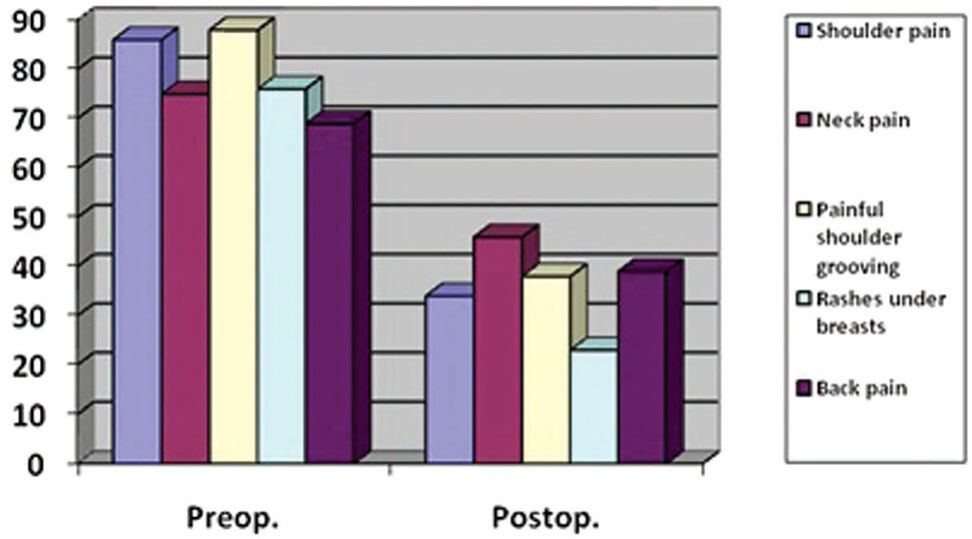
BREAST-Q symptom changes in breast reduction patients

All patients completed the BREAST-Q Reduction module. Scores were reported on a Likert scale from 0 to 100. The postoperative scores showed a statistically significant change from preoperative averages in symptom frequency. Symptoms including shoulder pain, neck pain, painful shoulder grooving from bra straps, inframammary rashes, and back pain were significantly reduced (*p* < 0.05) (Fig. 3).

BREAST-Q satisfaction scores demonstrated significant patient satisfaction across multiple domains, including breast appearance in clothing, how well breast size matched their body habitus, breast size, breast shape in a bra, comfort of bra fit, how the breasts hang, and how normal the breasts appeared postoperatively (*p* < 0.001) (Table 4).

**Table 4:** BREAST-Q satisfaction results

Questions for satisfaction	Preoperative (mean ± SD)	Postoperative (mean ± SD)
Breast appearance in clothes	59,0±1,8	79±2,4*
Breast size match to their body habitus	27,6±2,3	86±3,6*
Breast size	12,5±2,2	89±5,2*
Breast shape in a bra	18,0±2,7	92±4,4*
Comfort of bra fit	12,0±2,9	91±6,8*
How breasts hang	18,5±1,7	88±3,9*
How normal breasts appear	15,6±2,2	92±2,8*

*: (*p* < 0.001)

**Fig. 4:**
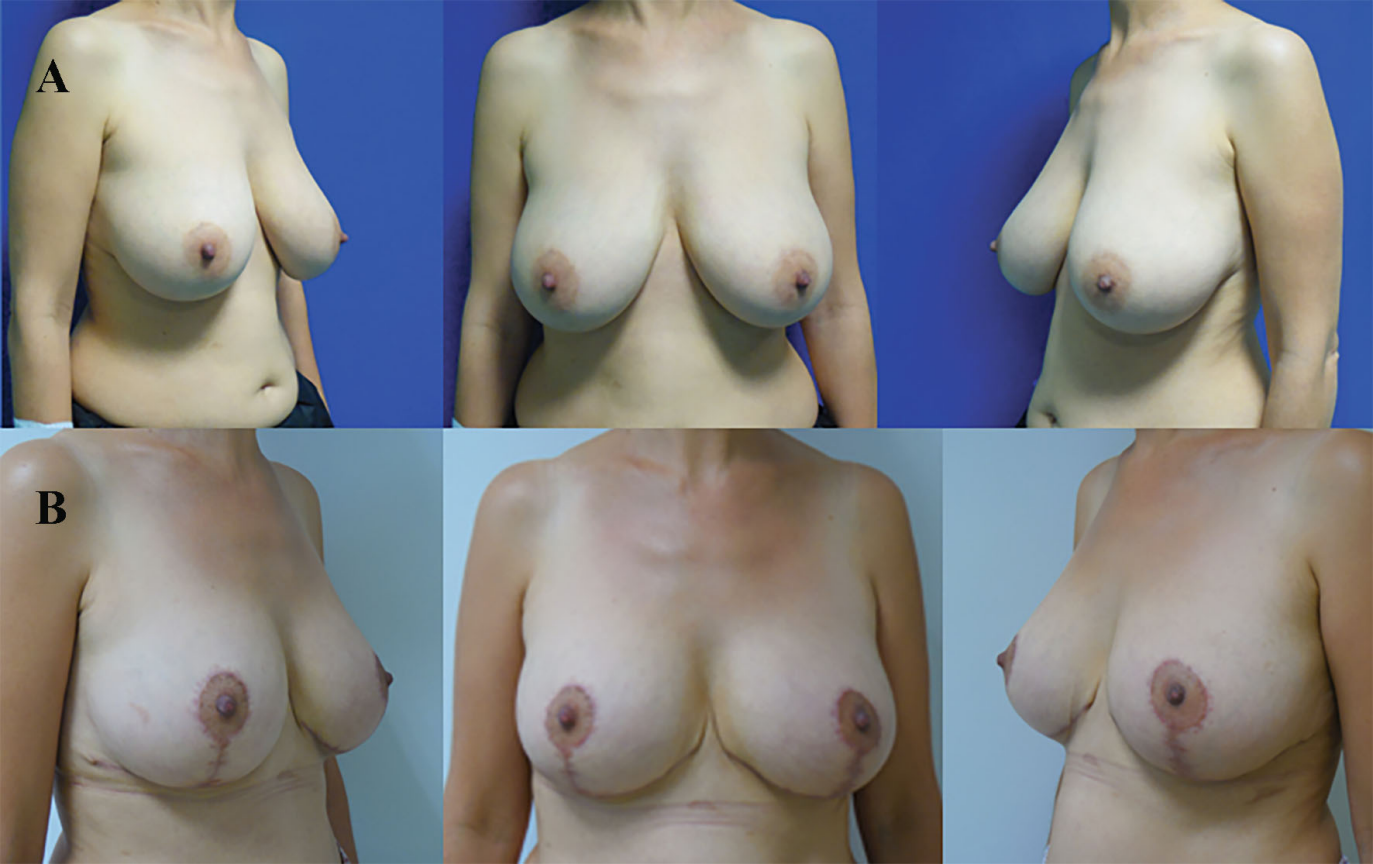
Patient example of early postoperative results. **A** Preoperative oblique and frontal views. **B** 6-month postoperative views

**Fig. 5:**
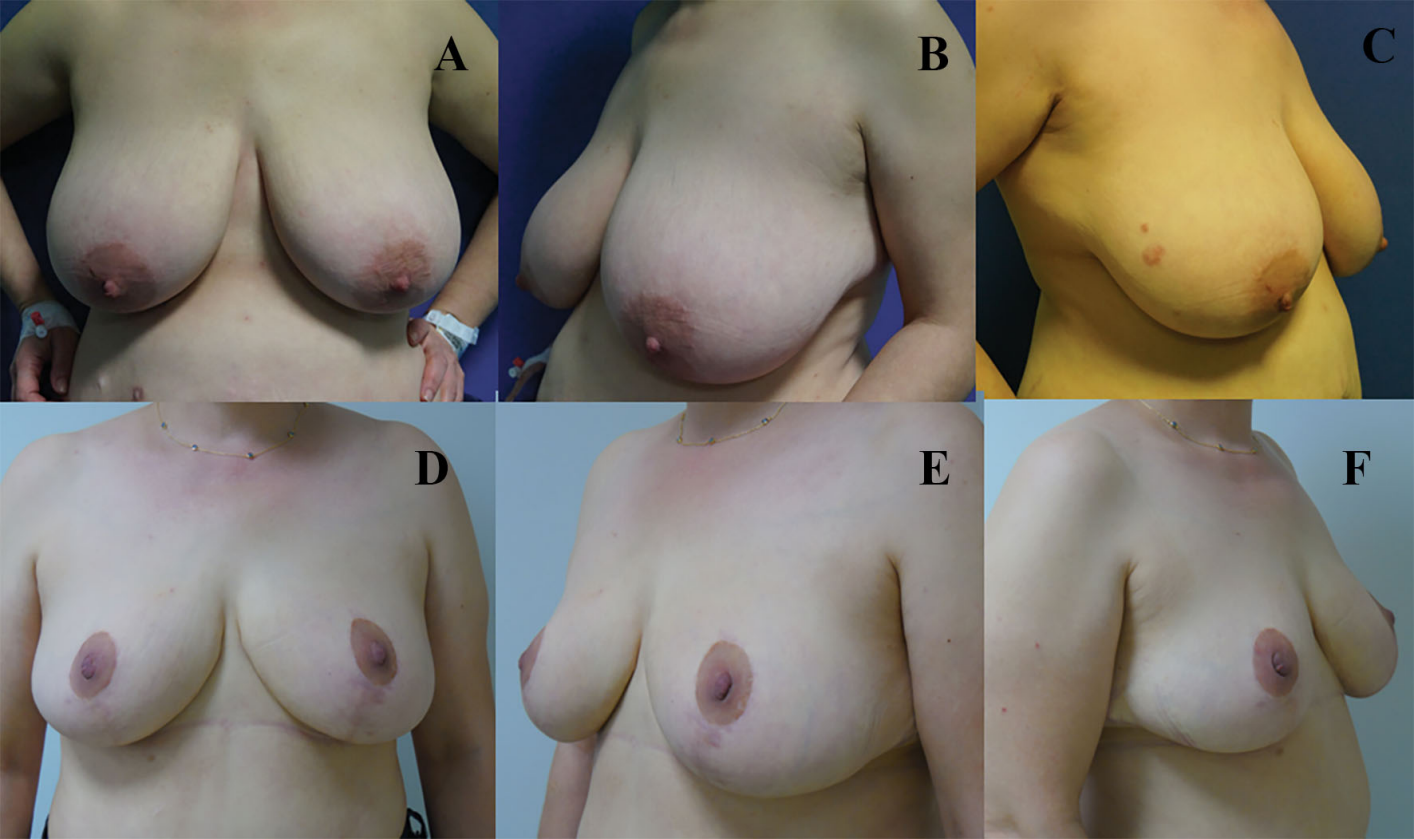
**A**, **B**, **C** Preoperative views of a patient. **D**, **E**, **F** 25-month postoperative appearances of the same patient

**Fig. 6:**
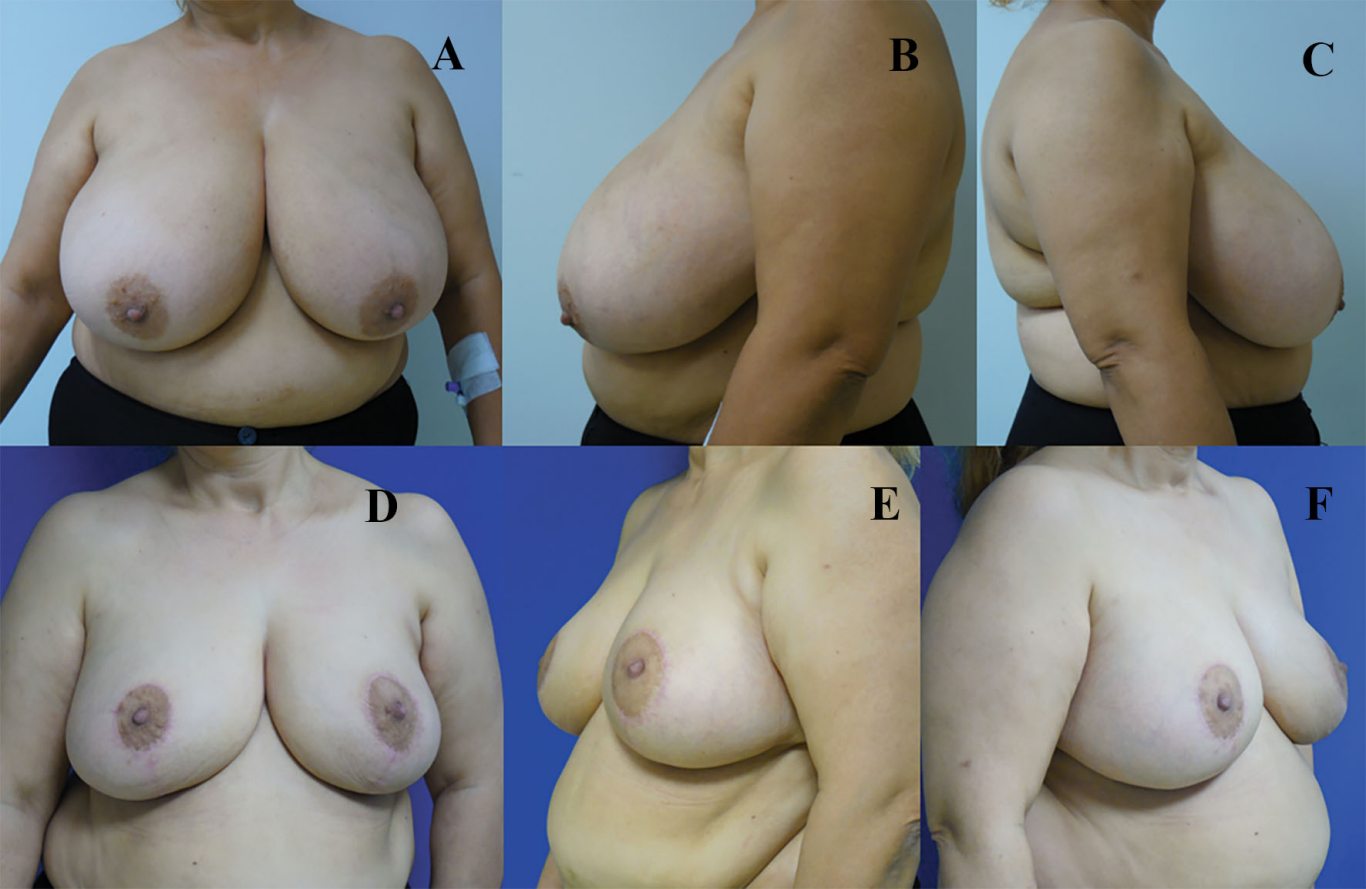
**A**, **B**, **C** Preoperative views of another patient; **D**, **E**, **F** 18-month postoperative views

Analysis of the final postoperative lateral photographs determined the number of breasts with a normal/perky upper pole, ptosis, or bottoming out (Table 5). The nipple was located at the apex of the breast mound in the majority of cases (122 breasts, 87%) (Figs. 4, 5 and 6). A perky upper pole appearance was noted in 19 breasts (13.5%), and no breasts showed true ptosis. A bottoming-out appearance was observed in 18 breasts (12.8%). Of these, 7 (38.8%) had a pedicle length greater than 15 cm.

**Table 5:** Breast view on the lateral photos

Breast appearance	Number	Rate (%)
Perky	19	13,5
Ptotic	0	0
Bottomed out	18	12,8
Normal	103	73,6

The revision rate was low. Two patients (1.4%) underwent scar revision due to dissatisfaction, and one patient (0.7%) requested a secondary reduction for perceived inadequate volume removal. One additional patient required a dog-ear excision on the mid-axillary line.

## Discussion

Although the inferior pedicle (IP) technique has been widely used in reduction mammaplasty with successful long-term results, it has been subject to criticism for its propensity to lead to pedicle sagging, loss of breast projection, deficient upper pole fullness, and the eventual development of a “bottoming out” deformity [1–4, 19, 20]. To overcome these drawbacks, creating a more predictable and stable breast shape is imperative. Consequently, numerous fixation and retention procedures have been advocated to secure the pedicle to the chest wall. Many modifications primarily suspend the inferior pedicle in its natural anatomical position over the pectoral muscle using dermal flaps, fascial slings, strong sutures, pedicle plications, pectoralis muscle loops, or alloplastic materials like acellular dermal matrix [7–15]. While these modifications have reported success in patients with macromastia in the early postoperative period, long-term outcomes have often shown some degree of inferior descent, resulting in bottoming-out deformity, an empty upper pole, and poor projection.

It appears that pedicle suspension alone is insufficient to maintain breast tissue in the upper and central regions, making sagging inevitable. The Mobile Inferior Pedicle (MIP) approach addresses this by converting the inferior-based pedicle into a chest wall-based flap through incision of its base down to the pectoral fascia. This modification grants the inferior pyramidal pedicle significant mobility by freeing it from its attachment at the inframammary fold. Advancing this lower central breast tissue like a biological implant into the central and upper pole addresses the deficiency in upper pole fullness, enhances breast projection, and reduces caudal bulk. Long-acting absorbable sutures placed between the pedicle dermis and the pectoral muscle provide robust stability, securing the pedicle in its new position and ensuring long-term maintenance of the breast shape. However, our late results showed some degree of bottoming out, so occurrence of bottoming out may increase with a longer follow-up.

Palmer and Taylor’s anatomical studies of the anterior chest wall demonstrated that the internal thoracic artery was the dominant vasculature supplying the breast region. This vessel forms interconnecting networks across all tissue planes with the posterior intercostal, lateral thoracic, thoraco-acromial, and transverse cervical arteries. Significant cutaneous perforators from these systems are concentrated along the perimeter of the pectoralis major muscle, the costal margin, and over the digitations of the serratus anterior muscle along the mid-axillary line. It is also demontrated that the dense concentration of large perforators along the inframammary crease, which originate from the anterior intercostal vessels within the fifth and sixth intercostal spaces [14]. As these anatomical findings are of critical importance for the design of pedicles in breast reduction procedures, in the MIP design, vascularity of NAC and inferior pedicle is additionally supported by means of protection of Würinger’s horizontal septum to compensate likely decrease in blood supply of them.

When the inferior pedicle is completely separated from the inframammary fold during dissection, which is theoretically capable of compromising blood perfusion of areolar area, vascularization of the pedicle and the nipple–areola complex (NAC) are derived from Würinger’s horizontal septum and the intercostal perforators of the fourth, fifth, and sixth ribs [15, 21]. Mojallal has described a breast reduction technique in which a posterosuperior pedicle was used with preservation of the anterior intercostal artery perforators. During glandular resection, fourth anterior intercostal arteries were preserved to enhance the reliability of the vascular supply to the superior pedicle. As anterior intercostal artery enters to the pedicle at the level of the mid-fourth and mid-fifth intercostal space, resection is ended at the inferior border of the pectoralis major muscle that is located at about 2–3 cm above the inframammary fold. Würinger’s horizontal septum is also protected in the technique [22].

Preservation mammoplasty concept is also arise from the anatomical basis of anterior intercostal arteries at the level of the fourth and fifth intercostal spaces. It aims to remove breast cancer mass and then to reshape the breast using the principles of breast reduction surgery to avoid a deformity. Reshaping and lifting techniques are planned with volume displacement or replacement, based on anatomical distributions of the internal thoracic artery, anterior and posterior intercostal arteries, lateral thoracic artery, and thoraco-acromial artery [23]. In the MIP technique, when inframammary fold is cut, some of the perforator arteries and subcutaneous capillaries are also dissected at the level of inframammary crease; however, main trunks of the fourth and fifth intercostal arteries are protected as strong sources of blood supply to the pedicle.

In our postoperative follow-up, there were two cases of nipple–areola necrosis, both in heavy smokers, and no permanent loss of NAC sensibility. Ren et al. recently reported a 1.8% NAC necrosis rate in a study of 114 patients undergoing inferior pedicle reduction [5]. Cang et al. performed a systematic review of 12 studies encompassing 618 patients who underwent the IP technique in 2025, finding NAC necrosis rates varying from 0 to 4% across studies [6]. In 2013, Antony also reported a 1% NAC necrosis rate in a study of 100 breast reductions [24]. Our observed rate of 1.4% NAC necrosis is consistent with these prior literature findings.

Although some studies report high rates of wound dehiscence and delayed healing due to excessive tension at the T-junction [2–4, 19, 20], the MIP technique reduced the occurrence of these complications. Friedman et al. reported that 27.6% of breasts had some degree of wound breakdown at the inverted-T portion; 7.7% required prolonged healing, and 1.2% required surgical intervention in their study of 918 breasts [4]. Ren et al. reported a 14% rate of wound opening after IP breast reduction in 114 patients [5]. A meta-analysis of 12 articles found rates for wound dehiscence, delayed healing, and skin necrosis ranging from 0 to 17.6%, 0–46.2%, and 0%, respectively [6]. In our study, the combined rate of delayed healing and wound dehiscence was 7.9%. By advancing the MIP, the lower pole over the inframammary fold is emptied, significantly reducing the weight-bearing forces on the midline of the lower pole, particularly at the T-junction. This facilitates a tension-free closure of the skin flaps, as they no longer bear the primary burden of supporting the breast mound. Consequently, the incidence of delayed healing and skin necrosis at the T-junction is reduced, and scars are less prone to hypertrophy. In this study, hypertrophic scarring developed in only two cases (1.4%), representing a low rate compared to previous studies [2–6].

O’Grady et al. studied complication rates in large (> 1000 g per breast) versus small (< 999 g per breast) inferior pedicle reductions. A review of 133 consecutive patients with a mean follow-up of 152 days found no statistically significant difference in rates of nipple necrosis, hematoma, seroma, delayed healing, infection, fat necrosis, cyst formation, or hypertrophic scarring between groups; however, the overall complication rate was reported as 56% for 266 breasts [3]. Compared to our overall complication rate of 22.8% with MIP, this suggests that the MIP technique may be associated with lower morbidity. While nipple necrosis rates differed (0.4% vs. 1.4%), complications, such as hematoma, seroma, and permanent loss of nipple sensation, were not observed in any MIP patient. Friedman et al. reported that 42% of 918 breasts had some complication, predominantly minor wound breakdown or fat necrosis [4]. Ren et al. demonstrated a 57% complication rate in 114 IP patients [5]. Cang et al. found overall complication rates between 6.6 and 49.7% following IP reduction [6]. Our observed rate of 22.8% is relatively lower and consistent with the lower end of the range reported in the literature [2–6].

Kulkarni et al. reported outcomes of reduction mammaplasty in adolescents, comparing Wise and vertical patterns [8]. Among 49 patients who underwent wise-pattern inferior pedicle reduction, major and minor complication rates were 2.0% and 20.4%, respectively. The most common complications were delayed wound healing (12.7%), wound dehiscence (6.1%), and fat necrosis (4.1%). The overall complication rate with MIP appears lower than this report, and their nipple necrosis rate of 2% was higher than our findings.

Ogunleye et al. examined complications in 39 patients who underwent the IP technique and compared them to 90 patients who underwent the superomedial pedicle technique [2]. While no statistically significant difference was found between the techniques, infection, wound dehiscence, and minor hematoma were the most common IP complications, with rates of 17.6%, 17.6%, and 8.8%, respectively. A total complication rate of 47.1% was encountered, with no nipple loss or major complications. The MIP complication rates appear significantly more acceptable.

Infection is a significant complication in breast reduction. Its occurrence varies in the literature based on technique, resection weight, BMI, age, and comorbidities [2–6, 19, 20, 24]. Friedman reported a 2.2% infection rate (affecting 4% of patients) in 918 breasts, with 2.5% of patients requiring surgical drainage [4]. Antony described a 1% infection rate [24], while Ren reported cellulitis in 21.9% of 114 IP patients [5]. Cang’s meta-analysis noted infection rates between 0 and 17.6% [6]. Our infection rate of 2.8% is significantly lower than many studies and falls within the reported range. Current literature reports overall complication rates for reduction mammaplasty ranging from 7.1 to 53% [2–4, 6, 19, 20], though definitive comparison is challenging due to differences in patient populations, resection weights, and follow-up times. In this study, we observed a lower overall complication rate than many previous studies on the inferior pedicle technique. These data provide strong evidence that the MIP procedure offers a safe alternative to traditional IP reduction mammaplasty.

Evaluation of BREAST-Q scores showed substantial and statistically significant improvement in all preoperative symptoms. Patients were highly satisfied with their changed breast appearance, reflected in very low revision rates: 1.4% for scar revision and 0.7% for re-reduction due to inadequate volume removal. Relief of breast-related symptoms and improved appearance, as captured by the BREAST-Q module, are consistent with outcomes reported in many other studies [2, 8, 17, 20].

Photographic evaluation using a two-dimensional system assessed changes in upper pole projection, overall breast projection, lower pole elevation, breast parenchymal ratio (upper-to-lower breast area on lateral images), and mound elevation. Patients undergoing MIP reduction demonstrated a significant increase in breast and upper pole projection. Significant lower pole and mean breast mound elevation were also observed, and the breast parenchymal ratio increased postoperatively. These outcomes suggest that the MIP technique effectively reduces volume while improving projection and aesthetics, providing a stable breast shape resistant to sagging in most patients. In all but 18 breasts, the nipple was located at the point of maximum projection. In those 18 cases (12.8%), bottoming out was observed, characterized by the nipple being over-elevated 1–2 cm above the point of maximum projection, creating an appearance of lower pole glandular excess.

However; when the pedicle is mobilized by cutting, it seems occurrence rate of some complications of IP reduces and some persists. Also, overall complication rate of this modification remains within the limits of the reported rate of complications of IP in the literature. Generally, while no universal and standard technique exists for reduction mammaplasty, selection of a surgical approach based on well-established neurovascular anatomy can improve surgical outcomes in those patients.

This study has several limitations that may introduce bias. It is a retrospective review of a relatively small patient cohort from a single surgeon and institution. Validation requires larger, multi-institutional prospective studies. Photographic evaluation was based on two-dimensional images and simple visual inspection, precluding statistical comparison of measurements. The wide range of follow-up is a major limitation, as the variable timing can directly impact the interpretation of outcomes like projection and bottoming out. Breast biomechanical properties, such as parenchymal firmness and skin elasticity, can vary considerably with age. The surgical technique may be heavily influenced by the inherent tissue characteristics of a specific age subgroup within the cohort. Therefore, the wide age range is a limitation of the study. Furthermore, as our outcomes are based solely on MIP patients without a control group undergoing traditional IP reduction, comparisons were inevitably made with historical literature, which is a methodological constraint.

In conclusion, the mobile inferior pedicle technique offers a safe and effective approach to reduction mammaplasty for patients with symptomatic macromastia. It provides theoretical advantages, including superior pedicle transposition, preservation of lactation potential and nipple sensation, a reliable blood supply, reduced tension at the T-junction, and a long-lasting, aesthetically pleasing breast shape with minimal sagging. Its learning curve is short for surgeons familiar with the inferior pedicle technique.

This procedure appears to mitigate the criticisms of the traditional IP method and may be a superior alternative for achieving an improved breast shape. Further research, ideally prospective controlled trials, is necessary to directly assess its performance against the standard IP reduction technique.
